# The Effectiveness of Cupping Therapy on Relieving Chronic Neck and Shoulder Pain: A Randomized Controlled Trial

**DOI:** 10.1155/2016/7358918

**Published:** 2016-03-17

**Authors:** Lee-Mei Chi, Li-Mei Lin, Chien-Lin Chen, Shu-Fang Wang, Hui-Ling Lai, Tai-Chu Peng

**Affiliations:** ^1^Institute of Medical Sciences, Tzu Chi University, Hualien 970, Taiwan; ^2^Department of Nursing, Tzu Chi University of Science and Technology, Hualien 970, Taiwan; ^3^Department of Nursing, Chang Gung University of Science and Technology, Taoyuan 333, Taiwan; ^4^Department of Chinese Medicine, Taipei Tzu Chi Hospital, New Taipei City 23142, Taiwan; ^5^School of Post-Baccalaureate Chinese Medicine, Tzu Chi University, Hualien 970, Taiwan; ^6^Chinese Lactation Consultant Association, Hualien 970, Taiwan; ^7^Department of Nursing, Tzu Chi University, Hualien 970, Taiwan

## Abstract

The research aimed to investigate the effectiveness of cupping therapy (CT) in changes on skin surface temperature (SST) for relieving chronic neck and shoulder pain (NSP) among community residents. A single-blind experimental design constituted of sixty subjects with self-perceived NSP. The subjects were randomly allocated to two groups. The cupping group received CT at SI 15, GB 21, and LI 15 acupuncture points, and the control group received no intervention. Pain was assessed using the SST, visual analog scale (VAS), and blood pressure (BP). The main results were SST of GB 21 acupuncture point raised from 30.6°C to 32.7°C and from 30.7°C to 30.6°C in the control group. Neck pain intensity (NPI) severity scores were reduced from 9.7 to 3.6 in the cupping group and from 9.7 to 9.5 in the control group. The SST and NPI differences between the groups were statistically significant (*P* < 0.001). One treatment of CT is shown to increase SST. In conjunction with the physiological effect the subjective experience of NSP is reduced in intensity. Further studies are required to improve the understanding and potential long-term effects of CT.

## 1. Introduction

Chronic neck and shoulder pain (NSP) is a type of musculoskeletal pain typically occurring in middle- and older-aged people [1–3]. The prevalence of NSP is approximately 16% to 78% among the general population [2–4]. The impact of chronic pain on the family includes social activities, life changes, emotional impact, and alteration of future plans [5].

Cupping therapy (CT) is a traditional Chinese medical (TCM) treatment which has been practiced for thousands of years. The World Health Organization's (WHO) definition of cupping is a therapeutic method (Code 5.3.2) involving the application of suction by creating a vacuum. This is typically done using fire in a cup or jar (Code 5.3.7) on the dermis of the affected part of the body [6].

In Taiwan, approximately 12.8% of the participants reported the use of cupping therapies in the past year [7]. The cupping mechanism constitutes creating a vacuum on the skin, with the ensuing negative pressure resulting in capillary rupture. This method is known as retained or dry cupping [8]. The skin of the localized area becomes flushed and may show petechiae and ecchymosis [9] or bruising, in which the duration is therapeutically beneficial [10]. Cupping has multiple therapeutic functions which include (1) warming the channels to remove cold, (2) promoting qi and blood circulation, (3) relieving swelling, (4) accelerating healing, (5) adjusting body temperature, (6) fibromyalgia [11], (7) stroke rehabilitation, hypertension, musculoskeletal pain, herpes zoster [8, 12], (8) facial paralysis, acne, and cervical spondylosis [13], and (9) alleviating pain [14], including chronic neck [15–17], shoulder pain [2], and low back pain [17, 18].

Traditional acupuncture points,* jianshongshu* (SI 15),* jianjing* (GB 21), and* jianju* (LI 15), have been suggested for improving NSP. The SI 15 point is positioned on the back, approximately 3 to 4 cm lateral to the lower border of the spinous process of the seventh cervical vertebra (dazhui). This point is associated with shoulder and back pain and coughing. The GB 21 is situated at the midpoint that connects the dazhui point (DU 14) and the acromion (the shoulder peak). It is primarily used to treat headaches, neck pain, stroke-induced speech impairment, and shoulder, back, and arm pain. The LI 15 point is located on the lateral side of the arm and on the deltoid muscle. It is the depressed area distal and anterior to the acromion when the arms are stretched outward or forward. This point is used to treat shoulder joint pain and hemiplegia [19].

The current literature remains sparse for studies on skin temperature differences at acupuncture points in relation to thermal effect of cupping therapy. Liu et al. showed that localized skin temperature increased [20, 21], while blood pressure decreased [22], after CT. It is suggested that these physiological responses to CT may be related to the positive therapeutic effect. Currently, due to the paucity of available research focusing on skin temperature changes due to CT, the potential effect and its relationship remains unclear. This study investigated the effectiveness of CT for relieving chronic NSP among community residents and the changes in skin surface temperature (SST).

## 2. Methods

### 2.1. Subjects

This study was a single-blind experimental design. Subjects with diagnosed and self-perceived chronic NSP were recruited in Hualien City, Taiwan, via advertising and e-mail from October 2012 to February 2013. This research was conducted in a nursing research laboratory at the Tzu Chi University of Science and Technology. The room temperature was controlled at 20 to 24°C and the humidity level was maintained at 60 to 70%. A Chinese traditional medicine nurse and traditional Chinese medical practitioner were also asked to verify the choice and location of the selected acupuncture points and the cupping treatment.

The inclusion criterion is as follows: (1) working at least 40 hours a week and (2) suffering work-related NSP continuously for at least 3 consecutive months with an intensity of at least 3 points on the visual analog scale (VAS, 0–10). Participants were excluded if the following exist: (1) infection, injury, or bleeding of the skin surrounding the area for cupping therapy, (2) neuropathy in the cervical spinal cord, (3) analgesic ingestion within 4 hrs preceding experiment, and (4) consumed coffee, tea, or any other caffeinated beverage within 4 hrs prior to the baseline measurement. Also, no tobacco products had been smoked for a minimum of 30 min before the baseline data were recorded.

### 2.2. Sample Size

In the pilot study (*n*  =  6) for NSP a statistically significant result between group difference of 1.18 (effect size = 0.81) using the VAS was found. Employing the Wilcoxon Mann-Whitney test (G power v 3.1.3) [23] to achieve a power of 0.8, with Cronbach's *α* value = 0.05 and an effect size of 0.80, the required size for each group is minimum of 27 subjects.

### 2.3. Randomization

**Figure 1:**
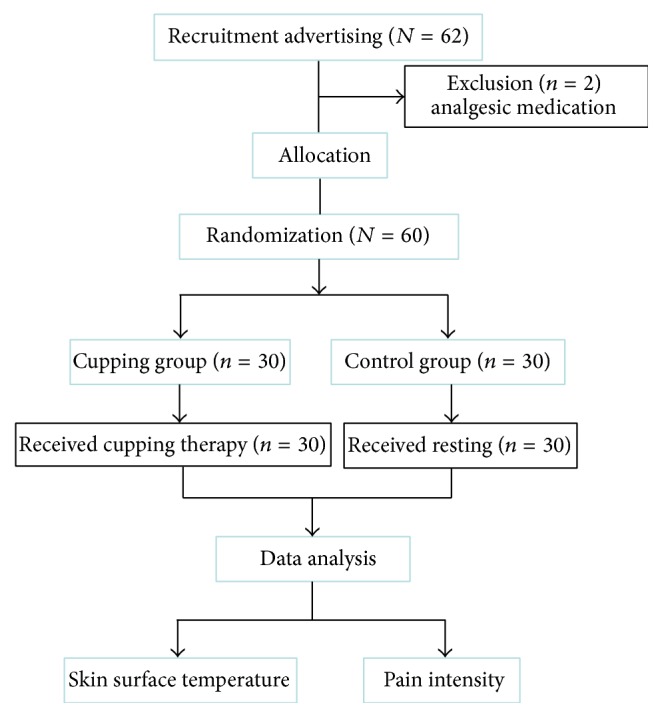
Flowchart of this study.

Subjects were assigned “cupping group” or “control group” based on random selection from sealed envelopes which had been sequence coded prior to study commencement. Neither the researcher nor the participants were aware of which group the participants would be assigned to.  Figure 1 displays the flowchart of the study.

This study was reviewed and approved by the Research Ethics Committee of the Buddhist Tzu Chi General Hospital (Registration number 101-60). Written consent was obtained from the participants prior to the start of the study. The objectives of the research were explained and the option to withdraw from the study at any time was made known.

### 2.4. Intervention

**Figure 2:**
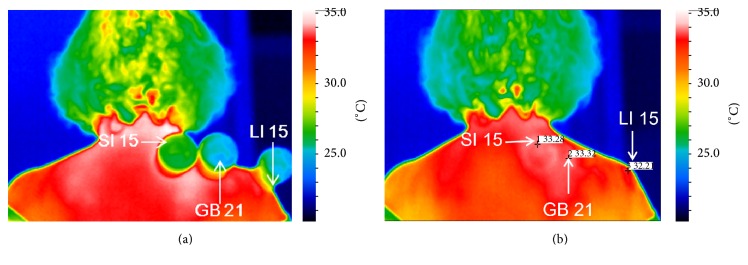
The skin surface temperature (°C) at SI 15, GB 21, and LI 15 acupuncture points displayed by infrared camera by cupping (a) and after cupping therapy (b).

The cupping group received fire CT at three acupuncture points, SI 15, GB 21, and LI 15. The medium size glass cup with diameter of 4 cm and volume of 260 mL (Cosmos International Supplies Co., Ltd., Taiwan) was used. Participants were asked to sit comfortably in a chair with both feet flat on the floor and expose their neck and shoulder regions. The cupping procedure is as follows: (1) an alcohol swab is ignited, (2) the burning swab is quickly placed inside the cup and withdrawn, (3) the cups are placed over the three acupuncture points, (4) the cups were then removed after 10 min [24], and (5) the same process was repeated for the same amount of time on the subject's left side (Figure 2(a)). The entire treatment totaled 20 minutes to treat both sides of the body.

Participants in the control group received resting for 20 min.

### 2.5. Outcomes

Participant characteristics included demographic data such as age, sex, and a brief medical history including past experience of cupping.

#### 2.5.1. Skin Surface Temperature (SST) and Blood Pressure (BP)

An infrared camera (FLIR ThermaCAM P25 HS system) was used to measure SST of the right SI 15, GB 21, and LI 15 acupuncture points (Figure 2(b)). Measurements were recorded for SST at 4 time points with a 5-minute interval between each measurement. The FLIR infrared camera is an infrared thermal detector, with 320 × 240 pixel geometric resolution of 76.800 pixels per picture. Measurements can be performed which range from 0 to 250°C ± 0.001°C. The data was transferred to a notebook computer using the ThermaCAM Researcher V.2.8 software (FLIR Systems Inc., Portland, Oregon, USA).

BP was measured using a mercury sphygmomanometer (Model S-300, standard sphygmomanometer, Taiwan) using the participants' right arm. BP was recorded both before and after intervention.

#### 2.5.2. Neck and Shoulder Pain Intensity

Pain was scored using VAS; a Likert scale was used for evaluating the subjective experience of pain intensity [25, 26]. The neck pain intensity test involved (1) leaning forward and backward, (2) rotating to the left and right, and (3) inclining to the left and right [27]. The shoulder pain intensity assessment involved (1) raising both arms, stretching the chest, and extending the arms backward to touch the back of the neck and (2) raising both arms upward, placing them against the ears, and placing the palms together [28]. The subjects were asked to select a point on the scale that most accurately reflected their level of pain before and then after the pain inducing movement [29].

### 2.6. Statistical Analysis

Data were analyzed using SPSS V.18.0 for Windows (SPSS Inc., Chicago, Illinois, USA). The univariate analysis of covariance (ANCOVA) was used to assess the level of NSP intensity. ANCOVA was used to assess the changes in the SST and BP, while adjusting the baseline for both groups. The Friedman test was conducted to evaluate the overall changes within each group. Wilcoxon test was used to compare the difference within groups. A *P* value of <0.05 was considered statistically significant.

## 3. Results

The study recruited a total of sixty-two participants and excluded two cases due to analgesic ingestion prior to the experiment. The participant gender representation within the study was female (91.7%; *n* = 55) and 8.3% male (*n* = 5). The subjects aged from 24 to 61 years with a median age of 43.6 ± 8 years. There were no significant differences between the cupping and control groups for subjects' gender and age at baseline (Table 1).

### 3.1. Skin Surface Temperature (SST) Changes

**Figure 3:**
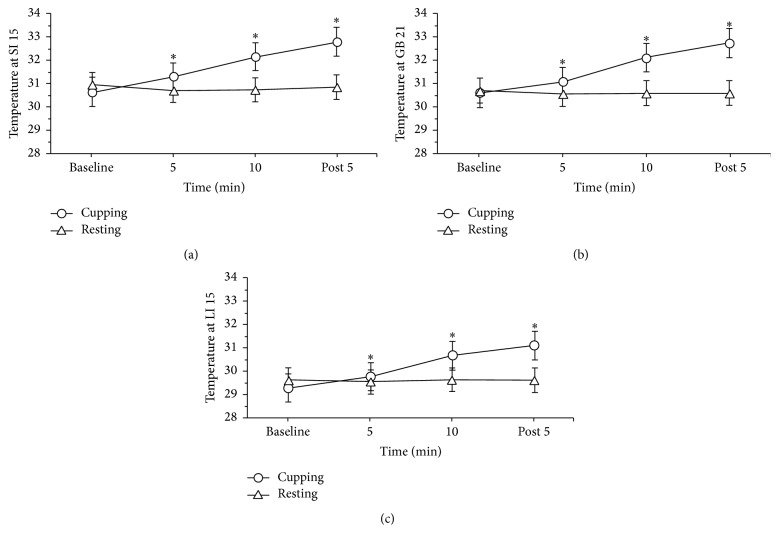
Change in SST (°C) at three acupuncture points during cupping therapy at 5-minute intervals. *∗*: difference between groups at SI 15 (a), GB 21 (b), and LI 15 (c) acupuncture points (*P* < 0.05).

The average temperatures at the SI 15 acupuncture point showed no significant differences between groups before CT. The SST at the SI 15 point increased to a peak of 32.8 ± 0.5°C at 5 minutes after CT. This temperature is significantly higher than the baseline (30.7 ± 0.5°C) (*P* < 0.01) (Table 2). The Friedman tests revealed that, from baseline to 5 minutes after cessation of treatment, CT acts to increase the SST of SI 15 (*P* < 0.01). During the resting period for the control group, the SI 15 temperature showed no significant difference from baseline (*P* > 0.05) (Figure 3(a)).

The average temperatures at the GB 21 acupuncture point showed no significant differences between groups before CT. The SST of the GB 21 point gradually increased to a peak of 32.7 ± 0.6°C after 5-minute CT. This value is significantly higher than the baseline of 30.6 ± 0.5°C (*P* < 0.01). The Friedman tests revealed that, from baseline to 5 minutes after CT, the SST of GB 21 remained elevated (*P* < 0.01). The control group, during the resting period, showed a gradual decrease in temperature to 30.6 ± 0.5°C at 15 minutes at the GB 21 acupuncture point. There were no significant differences from baseline (30.7 ± 0.4°C) (*P* > 0.05) (Figure 3(b)) for GB 21 within the control group.

The SST of the LI 15 was 29.4 ± 0.4°C at baseline within the cupping group and 29.7 ± 0.4°C within the control group (*P* > 0.05). The SST of the LI 15 point increased to 31.1 ± 0.8°C at 5 minutes after CT, which is significantly higher than baseline (*P* < 0.01). The Friedman test supports the within group results, which show that, from baseline to 5 minutes after cessation of treatment, SST remains elevated at LI 15 (*P* < 0.01). The ANCOVA test indicates significant differences between the groups at each time point for GB 21, SI 15, and LI 15 acupuncture points (*P* < 0.05) (Figure 3(c)). It is important to note that the results for LI 15 show lower temperatures. This is due to the distance from the acupuncture point to the lens of the infrared camera.

### 3.2. BP Changes

The systemic blood pressure (SBP) decreased from 117.7 ± 2.9 mmHg to 111.8  ±  2.3 mmHg, in the cupping group (*P* = 0.003). The control group also showed slight reduction from 113.8 ± 3.0 mmHg to 109.7 ± 3.1 mmHg (*P* = 0.117). There was no significant difference between the two groups; however cupping appears to have some influence on the SBP.

### 3.3. Pain Intensity Changes

**Figure 4:**
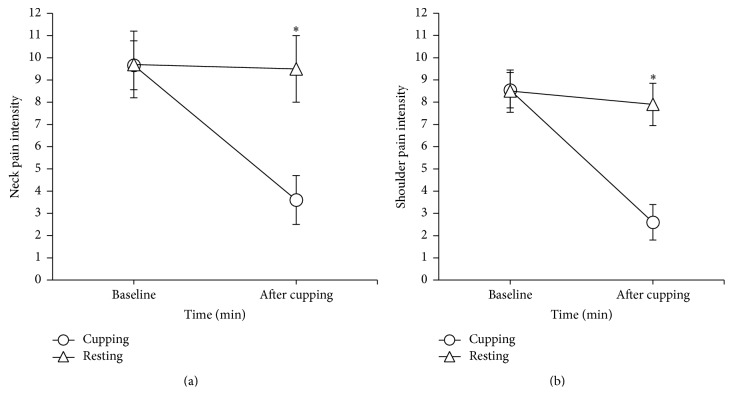
Visual analog scale (mean ± SEM) of subjects with chronic neck pain (a) and chronic shoulder pain (b). *∗*: univariate analysis of covariance (ANCOVA) was used to compare groups' difference after adjustment of baseline differences (*P* <0.05).

At baseline, the VAS of neck pain intensity (NPI) was 9.7 ± 1.6 in the cupping group and 9.7 ± 1.6 in the control group. The posttreatment NPI decreased by 6.1 in the cupping group and decreased by 0.2 in the control group (Figure 4(a)). The ANCOVA test demonstrated significant differences between the groups (*P* < 0.001).

The VAS of shoulder pain intensity (SPI) was 8.5 ± 0.9 for the cupping group at the baseline and 8.5 ± 0.9 in the control group. The posttreatment SPI decreased by 5.9 in the cupping group and decreased by 0.6 in the control group (Figure 4(b)). The difference between the groups was statistically significant (*P* <  0.001).

## 4. Discussion

The CT therapeutic method can cause vasodilatation and stimulate blood circulation to increase metabolism and accelerate the elimination of waste and toxins from the body. This effect acts to improve physical function [30] and affect BP [22]. Xu et al. demonstrated changes in skin temperature in the cupping area before and after cupping. When the cup was removed, 10 minutes after cupping, the skin temperature in the cupping area was elevated compared to the control area and showed significant difference [21]. Al-Rubaye also showed immediate clinical changes after cupping which included the sensation of increased warmth on the skin surface [22]. Similarly, Liu et al. showed that blood flow to the skin of the back in healthy humans on acupuncture points increased immediately following removal of the cup [20]. After CT, several other immediate signs of the therapeutic method may be observed and are dependent on the modality in use. Cupping increases blood flow to the cupped region (hyperemia); the subject experiences warmth as a result of vasodilatation. Due to vasodilatation and edema, histological changes are readily observable at the skin surface. After cupping effects often include erythema, edema, and ecchymosis in a variety of circular arrangements [31].

Cupping increased SST in this study. The results showed that SST at GB 21 was elevated from 30.6 to 32.1°C during the cupping period and increased after removal of the cup. Similarly, both SI 15 and GB 21 acupuncture points showed increased SST (2.1°C) after cup removal at the 5 min interval. At the LI 15 acupuncture point SST was elevated by 1.7°C.

The study outcome supports the efficacy of CT as a complementary therapy for treating NSP. The results indicate that CT provides significant and effective relief of NSP compared to the control.

Yuan et al. conducted a systematic review and meta-analysis of traditional Chinese medicine for neck pain and low back pain. It was suggested that cupping may be more effective than medications for treatment of chronic neck or lower back pain [17]. Lauche et al. targeted 50 participants with nonspecific neck pain and implemented 10 to 15 min of cupping therapy on the lower trapezius muscle. Their results showed that, at rest and during movement, the pain level on the VAS (0–10) decreased by 1.79 and 1.97, after cupping, respectively [16]. Kim et al. found that 6 sessions of cupping therapy (wet and dry) on neck pain acupuncture points in 40 patients were more effective than the use of a heating pad [15]. The German study of Lauche et al. found that home-based CT was more effective than progressive muscle relaxation in patients with chronic neck pain. The pain reduction effect remained evident at the one week after intervention interval [32].

Huang et al. employed cupping therapy around the neck and shoulder regions, combined with acupuncture and massage. This treatment was implemented once a day to comprise one session. A full course of treatment entails five sessions and a total of four courses were conducted for the experiment. Their results illustrated that this regimen could significantly reduce shoulder pain [33]. The current study used ANCOVA to assess the level of NPI and SPI. The baseline was adjusted in both groups to control for the potential bias when using VAS. This allows for a more reliable assessment of the CT effect.

In the current study, no participants experienced localized skin burns or adverse reactions in the treatment regions. Two participants in the cupping group reported mild low back pain related to the seated position. In the systematic review by Yuan et al. no serious or life-threatening side effects were noted [17]. The majority of adverse effects are related to wet cupping therapy, which results in (1) skin laceration, (2) whole body itching, (3) pain at the cupping sites, (4) generalized body ache [15], (5) factitious panniculitis, and (6) iron deficiency anemia [9]. Dry cupping, by comparison, is a safe and effective treatment modality for NPI and SPI.

CT is often used to treat pain, such as low back pain, fibromyalgia, shoulder pain, chronic nonspecific neck pain, cardiovascular diseases, angina, arthritis, and high blood pressure. The clinical evidence of CT is minimal [34]. Findings from this study strongly suggest that CT is effective for relieving pain, with no adverse effects. CT has the potential to eliminate reliance on analgesics and reduce health care costs.

Limitations of the study were primarily based on the limited availability of the participants. As participants were available for only one session, follow-up or multiple sessions were not possible. Improving the validity and reliability of this research requires (1) increasing the number of therapy sessions, (2) enlarging the sample size, (3) achieving equal representation of both sexes, and (4) age distribution.

## 5. Conclusion

Chronic NSP is a common problem in adults. CT is one of many effective treatments in traditional Chinese medicine. CT is used worldwide, as it is easy to learn and has few side effects. In this study, one treatment of CT is shown to increase SST and reduce SBP. In conjunction with the physiological effects, the subjective experience of NSP is reduced. CT mimics an analgesic effect which has no known negative side effects and may be considered safe. However, further studies are required to improve the understanding and potential long-term effects of CT.

## Figures and Tables

**Table 1:** Group demographic characteristics. Categorical variables: Chi-square test. Continuous variables: Mann-Whitney *U* test.

Variables	Cupping *n* = 30	Resting *n* = 30	*P*
Gender (%)			0.640
Male	3 (10.0)	2 (6.7)	
Female	27 (90.0)	28 (93.3)	
Age (mean ± SD)	43.6 ± 8.0	42.5 ± 7.4	0.486

**Table 2:** Changes in SST at three acupuncture points between groups at 5-minute intervals. p 5th min: the 5th min of rest after cupping therapy. Note: ^+^ANCOVA was used to compare groups' difference after adjustment of baseline differences. ^++^Friedman test was used to compare the difference within group. ^*∗*^ *P* < 0.05.

Measurement indices	Mean (SEM)				Friedman test	
	Baseline	5 min	10 min	p 5th min	*χ* ^2^	*P* ^++^
SI 15						
Cupping	30.68 (0.51)	31.33 (0.45)	32.18 (0.46)	32.82 (0.53)	14.040	0.003^*∗*^
Resting	30.99 (0.57)	30.72 (0.58)	30.78 (0.57)	30.89 (0.59)	3.367	0.338
*F*	—	11.915	32.684	48.949		
*P* ^+^	—	0.011^*∗*^	0.001^*∗*^	0.001^*∗*^		
GB 21						
Cupping	30.62 (0.50)	31.09 (0.61)	32.08 (0.71)	32.72 (0.62)	14.040	0.003^*∗*^
Resting	30.71 (0.42)	30.57 (0.50)	30.61 (0.47)	30.60 (0.45)	1.653	0.647
*F*	—	16.930	8.548	22.729		
*P* ^+^	—	0.004^*∗*^	0.022^*∗*^	0.002^*∗*^		
LI 15						
Cupping	29.39 (0.39)	29.78 (0.42)	30.70 (0.47)	31.12 (0.78)	11.880	0.008^*∗*^
Resting	29.65 (0.37)	29.56 (0.40)	29.65 (0.43)	29.64 (0.46)	0.120	0.989
*F*	—	9.007	28.726	24.828		
*P* ^+^	—	0.020^*∗*^	0.001^*∗*^	0.002^*∗*^		
